# Targeted Genome-Wide Enrichment of Functional Regions

**DOI:** 10.1371/journal.pone.0011138

**Published:** 2010-06-16

**Authors:** Periannan Senapathy, Ashwini Bhasi, Jeffrey Mattox, Perundurai S. Dhandapany, Sakthivel Sadayappan

**Affiliations:** 1 Department of Human Genetics, Genome Technologies, LLC., Madison, Wisconsin, United States of America; 2 Department of Bioinformatics, International Center for Advanced Genomics and Proteomics, Chennai, India; 3 Departments of Pediatrics and Genetics and Genomic Sciences and the Child Health and Development Institute, Center for Molecular Cardiology, Mount Sinai School of Medicine, New York, New York United States of America; 4 Department of Cell and Molecular Physiology, Stritch School of Medicine, Loyola University Chicago, Maywood, Illinois, United States of America; Institute of Preventive Medicine, Denmark

## Abstract

Only a small fraction of large genomes such as that of the human contains the functional regions such as the exons, promoters, and polyA sites. A platform technique for selective enrichment of functional genomic regions will enable several next-generation sequencing applications that include the discovery of causal mutations for disease and drug response. Here, we describe a powerful platform technique, termed “functional genomic fingerprinting” (FGF), for the multiplexed genomewide isolation and analysis of targeted regions such as the exome, promoterome, or exon splice enhancers. The technique employs a fixed part of a uniquely designed Fixed-Randomized primer, while the randomized part contains all the possible sequence permutations. The Fixed-Randomized primers bind with full sequence complementarity at multiple sites where the fixed sequence (such as the splice signals) occurs within the genome, and multiplex amplify many regions bounded by the fixed sequences (e.g., exons). Notably, validation of this technique using cardiac myosin binding protein-C (*MYBPC3*) gene as an example strongly supports the application and efficacy of this method. Further, assisted by genomewide computational analyses of such sequences, the FGF technique may provide a unique platform for high-throughput sample production and analysis of targeted genomic regions by the next-generation sequencing techniques, with powerful applications in discovering disease and drug response genes.

## Introduction

The large scale sequencing of thousands of genomes from disease cohorts by the next generation sequencing (NGS) techniques are expected to uncover causal gene mutations [1]–[5]. As the large-scale NGS capabilities and logistics are becoming established, target enrichment of genomes will be a boon for specific applications involving massive parallel scanning of targeted genomic regions [6]. The scalable technique we describe in this report, termed Functional Genomic Fingerprinting (FGF), is capable of selectively amplifying and screening any genomic functional regions bounded by short repeated sequences such as the exons that are bordered by splice signals, or regions surrounding the regulatory elements (e.g., promoters and polyA sites) within a genome. Whole or selective subsets of exomes, promoteromes or sequences such as the lariat sites or exon-splice enhancers can be isolated by this technique. As considerable fraction of causal mutations is located within genetic regulatory regions [7]–[13], FGF may provide a powerful technique for causal mutation discovery.

In this paper, we describe the computational analysis of the human genome for testing the feasibility of the FGF method. We then apply the FGF method to a small subset of exons in the cardiac myosin binding protein-C gene (*MYBPC3*) for demonstrating that FGF is capable of multiplex amplifying exons from the genome. Automation of FGF method might be a powerful target enrichment strategy for selective genomic functional regions, which may have potential applications in medical, agricultural and biotechnology fields. If this technique is demonstrated, then it should be currently feasible to scale it to sequence selective functional regions of the genome, such as the exome or promoterome, on NGS platforms, and compare them between cohorts of 1000 patient versus 1000 normal individuals, enabling the identification of causal genes. As a targeted polymerase chain reaction (PCR) based technique that largely avoids non-specificity, FGF may be a viable and cost-effective alternative to array and solution based hybridization enrichment technologies.

## Results

### Design of Fixed-Randomized (FR) primers

**Figure 1:**
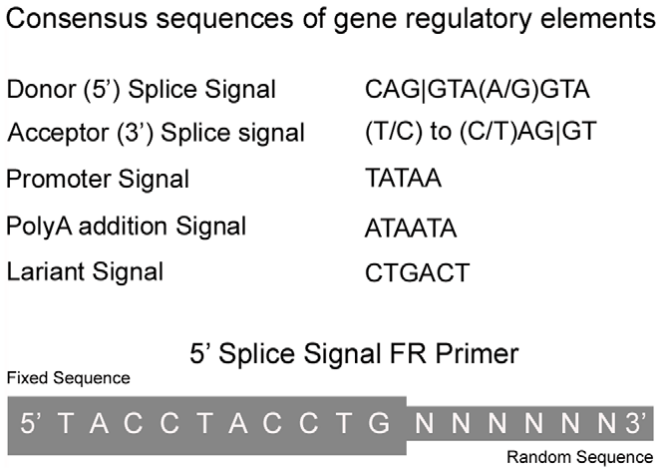
The consensus sequences of genetic regulatory elements. The consensus sequences of the gene regulatory regions are usually 6–12 bases long. Since the donor (5') splice signal reads into the intron, its complementary sequence is used to design a primer that will read into and amplify the exon. In this example, the fixed sequence is on the 5' end, and the randomized sequence is on the 3' end of the FR primer.

**Figure 2:**
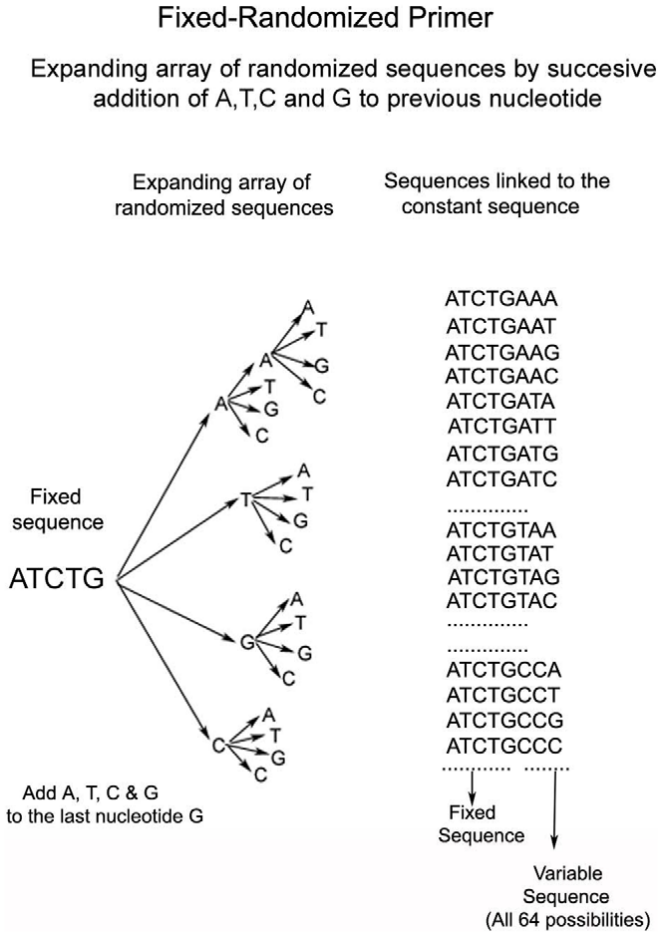
Design of an FR primer. To a core fixed sequence (ATCTG), a series of Ns (A, T, G and C) are added in equimolar concentrations at each step of the oligo-nucleotide synthesis. This process generates all possible sequences of length n, when n bases are randomized, such that each variable sequence is attached to the end of the fixed sequence.

The exceptionality of FGF lies in its ability to focus on the sequences of the functional regions of a genome such as the promoter, polyA site, branch point site, and the 5′ and 3′ splice-junction signals. This ability largely depends upon the unique design of the primers employed. The consensus sequences of regulatory signals in eukaryotic genes are typically short, approximately 6–12 bases in length [14] (Figure 1). FGF exploits such short-sequence feature of the regulatory sequences with Fixed-Randomized (FR) primers, which are each made up of one fixed (F) and one randomized (R) sequence part. The F sequence consists of 6–12 bases that are complementary to a target consensus sequence. The R part contains all possible sequence combinations, allowing for complete complementary base pairing over the entire length of the FR primers at multiple locations wherever the fixed sequence occurs in a template DNA. These FR primers are thus uniquely designed to bind at multiple locations of a given regulatory sequence throughout the genome, while providing a full, ideal length primer capable of binding at each of these locations. The power of the FR primers is that the correct fully complementary sequence primer will selectively bind at each of the target site, as only that primer species will be able to bind at a high melting temperature (Tm) used in the PCR reaction. Thus an FR primer set contains a series of primers, each of which contains the same F portion and a variable R portion of a given length with all possible sequence variations. Figure 2 depicts the design and the synthesis of an FR primer set with a five-base fixed sequence and a three-base variable sequence. After three serial additions of all the four bases (A, T, G or C) at each synthetic step to a fixed five base sequence, the resultant FR primer set contains all of the possible 64 triplets attached to the fixed ATCTG sequence.

**Figure 3:**
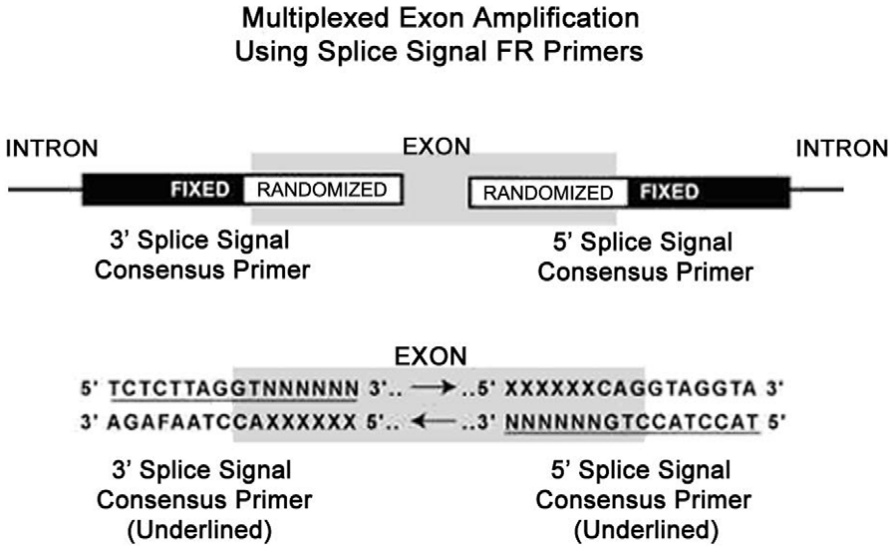
Multiplexed amplification of exons by splice signal FR primers. The FR primers for the donor (5′ splice signal) and the acceptor (3′ splice signal) splice sequences bind the exons with complementary base pairing over the entire length of the primers, by virtue of the presence of all the possible sequences within the randomized sequence portion of the FR primer. Only the specific primer molecule from the FR primer population is expected to bind selectively with full complementarity at the fixed sequence target site at a high Tm condition. The FR primers amplify multiple exons since they are capable of binding many exons within the human genome with complete sequence complementarity, wherever the fixed sequence binds.

The basic principles of the FGF technique for multiplexed amplification of exons in the human genome are summarized in Figure 3. Note that the donor and acceptor splice signal sequences of an exon are set as the F portions of two different FR primers. Hence selected human genomic DNA fragments are amplified with this pair of ‘splice-signal’ FR primers. In practice, this scheme produces multiplexed amplification of many exons from different genes in the human genome and the amplified products can be easily detected by electrophoresis. Genetic variants can be identified with comparative FGF in which FGF is conducted with both a test genome (i.e. from an individual with a disease or genetic trait of interest) and normal genomic DNA, using a series of ‘splice-signal’ FR primers with varying sequences, and the resultant polymerase chain reaction (PCR)-amplified fragments from each are displayed side by side. Differing fragments can then be sequenced to identify specific mutations.

### Computer modeling of multiplexed exon amplification of the human genome

We conducted a detailed computer simulation, wherein the whole human genome sequence was PCR amplified with the 5′ and 3′ splice signal FR primers. The FGF ePCR software program searches both strands of the genomic sequence and reports all the locations of the forward and reverse primer sequences. It then reports the locations of the fragments (amplicons) under a specified length that are bounded by the forward and reverse primers. The program then identifies whether each fragment represents an exact exon, part of an exon, intronic or intergenic sequence by comparing the amplicons with the data of annotated genes in Refseq Human Genome Build (NCBI 36.1).

The length of the fixed primer sequence determines the probability and thus the frequency of its occurrence within a genome. We conducted a simulation of PCR amplification of the human genome with FR primers containing different numbers of fixed and randomized bases. For the size of the human genome (3.2 billion base pairs), a given sequence of 15–16 bases in length occurred once, on average. Our results indicated that a fixed length of 13 or more bases in the FR primer pairs generated either none or only one or two fragments. They showed that a fixed length of 9–12 bases in the FR primer pairs generated many fragments in the desired range of 10–100 fragments, ideal for fingerprint analysis (results not shown). These primer lengths were also the length range of splice signal consensus sequences, and were ideal for amplifying exons from the human genome when used in conjunction with a randomized sequence length of approximately 6–8 bases. Furthermore, the results indicated that approximately 60–75% of the amplified fragments matched or overlapped with exons. However, given that the majority of human genes annotated in RefSeq are computer predicted (∼6,000 genes are experimentally validated and ∼21,000 genes are predicted) [15], the fraction of ePCR exons matching the real exons will be higher if the number of genes in the genome will be found to be higher in the future. In addition, the probability that splice signals border true exons is higher in smaller genomes, such as those of the Arabidopsis, Drosophila and Plasmodium, because the fraction of intron sequences within these genomes is considerably less [16]; therefore the vast majority of the fragments amplified by FGF primers must be real exons.

The length of the fixed primer sequence determines the probability and thus the frequency of its occurrence within a genome. While the primer lengths between 9–12 bases will generate between 10–100 fragments that is ideal for fingerprint analysis within the human genome (3.2 billion base pairs), a higher coverage of exons is desired for NGS applications. This should be feasible by decreasing the fixed length of the primers by even one or two bases, and/or by incorporating variable bases within the fixed sequence of the FR primer as appropriate to the functional element.

### Feasibility testing of FGF

**Figure 4:**
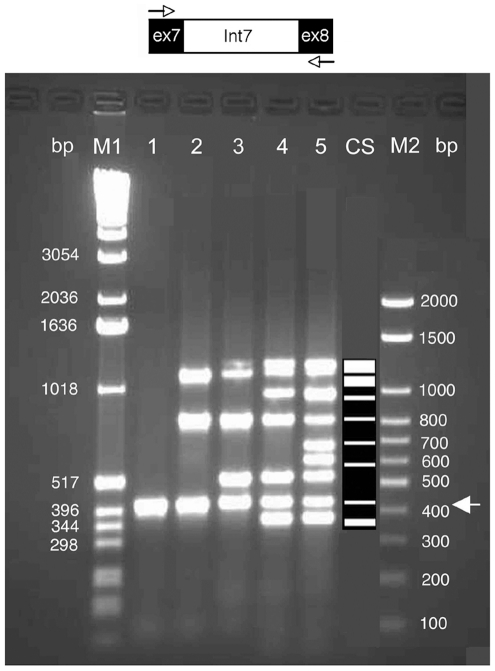
Multiplex genome-wide exon amplification based on MYBPC3 gene exons 7 and 8. The human genomic DNA was PCR-amplified under standard conditions at 58°C with primers designed from the donor (5′) splice signal sequence (from exon 7) and the acceptor (3′) splice signal sequence (from exon 8) of the MYBPC3 gene. It was also amplified by the same FR primer pairs with decreasing number of fixed bases and increasing number of random bases (Ns) as shown in Table 1. The expected fragment (438 bases for the combined exon 7, intron 7 and exon 8) is present in all the lanes, and the number of fragments amplified increased with increasing Ns in the FR primers. M1 & M2 are marker lanes. Lane CS shows the computer simulated exon fingerprint obtained with the same primers used for lane 5, with four bases removed from the 5′ end of the forward primer and three bases removed from the 5′ end of the reverse primer (see text).

The objective of this study was to test whether the FR primers would amplify exons from the human genomic DNA according to the expected results predicted by the FGF technique. We used exons 7 and 8 of the *MYBPC3* gene (containing 35 exons), which were also examined by ePCR computer simulation (see above). MYBPC3 is a thick filament associated protein in the cardiac sarcomere which contributes 2% of the total heart mass. Mutations in *MYBPC3* can cause hypertrophic cardiomyopathy that is characterized by cardiac hypertrophy, myocyte disarray, contractile dysfunction and sudden death. Importantly, mutations in this gene have been linked to hypertrophic cardiomyopathy in more than 60 million people worldwide. We defined a series of FR primers based on the donor and the acceptor splice signal sequences at the extreme ends of these exons, such that the specified number of bases at the 3′ end of these primers were replaced by a series of random nucleotides (Figure 4). The randomized sequence at the 3′ end of the primer is expected to afford the specific selection of the correct complementary sequence from among the population of primer sequences, which contain all of the possible sequences of the length of the randomized sequence during PCR amplification. Further systematic studies will delineate the difference in using the fixed sequence at the 5′ end or the 3′ end of the FR primer. While the full-length primer pair should amplify only the expected fragment, encompassing the two exons and the intervening intron (exon 7 – intron 7 – exon 8), the FR primer pairs should amplify increasing numbers of exons as the number of fixed bases is decreased and the number of randomized bases (Ns) is increased.

To test these predictions, human genomic DNA was PCR amplified with the full-length primers and the accompanying series of splice signal FR primers (sequences are shown in Table 1). Note that the concentrations of the primers used were increased progressively as the number of Ns increased to compensate for the degeneracy of the 3′ bases. The PCR products were resolved by agarose gel electrophoresis (Figure 4, lanes 1–5). As expected, the full-length primer pair, in which the primer was 100% complementary to the donor sequence of exon 7 and the acceptor sequence of exon 8 with no Ns, resulted in the expected single, 438 base PCR product (lane 1). When 2N FR primers were used (lane 2), such that the primer preparation contained all 16 possible *di-nucleotide* sequences at their 3′ ends, amplification of the exon 7–8 fragment was observed together with amplification of two additional fragments. The two new fragments presumably correspond to unknown exon regions of other genes. Likewise, when 4N FR primers were used (lane 3), such that primers contained all the 256 possible tetra-nucleotides at their 3′ ends, four additional exons were seen in addition to the product seen with the full length fixed primers. Further increasing the number of random nucleotides (5Ns and 6Ns) resulted in more bands (7 and 9 fragments, respectively). Importantly, the expected exon fragment amplified by the full-length primer pair was present in all of the lanes, and the fragments generated by each FR primer set always represented a subset of fragments generated by FR primers with a greater number of Ns. Thus by decreasing the number of fixed primer bases and increasing the number of Ns in an FR primer set, one can amplify an increasing number of new exon fragments from other genomic regions.

**Table 1:** FR Primers used in the amplification experiments shown in Figure 4.

Lane	Forward primer /Reverse primer	Number of random nucleotides	Primer per 25 µl reaction	Expected (used) fold excess primers^a^
1	**ACCCAG**AGGCCATGGGCAC/ **CTATC**ACTATGGAGAGGGACC	0 N	12.5 pmol	1
2	**ACCCAG**AGGCCATGGGCNN/ **CTATCA**CTATGGAGAGGGANN	2 N	200 pmol	16 (16)
3	**ACCCAG**AGGCCATGGNNNN/ **CTATCA**CTATGGAGAGGNNNN	4 N	1400 pmol	256 (116)
4	**ACCCAG**AGGCCATGNNNNN/ **CTATCA**CTATGGAGAGNNNNN	5 N	2000 pmol	625 (160)
5	**ACCCAG**AGGCCATNNNNNN/ **CTATCA**CTATGGAGANNNNNN	6 N	2000 pmol	1296 (160)

a Excess FR primers required theoretically for each specific primer sequence within the primer population to be the same as in the standard PCR reaction compared to the actual excess used in the experiment.

We employed our computational ePCR FGF simulation (see above) to predict the exon amplicons generated by the splice signal FR primer pairs shown in Table 1. We also developed a computer program for simulating the electrophoretic gel pattern of the electronic PCR amplified fragments. This gel pattern was compared with the actual gel image of the amplified fragments. There was less number of fragments predicted by the simulation than those in the experimental gel when we used the FR primers with 6Ns on the 3′ end (Table 1). However, it was possible that, because the FR primers used in the experiment were long (5′ primer: 19 bases; 3′ primer: 21 bases), some of the bases on the 5′ end of the primers may not have bound with the template DNA. When we removed four bases from the 5′ end of the forward FR primer and 3 bases from the 5′ end of the reverse FR primer (the primer sequences used were: 5′ splice signal: AGAGGCCATNNNNNN; 3′ splice signal: TCACTATGGAGANNNNNN), the match between the simulated and experimental fragments was the maximum. Between the 11 computer predicted fragments (lane 6) and 10 experimentally amplified fragments (lane 5), eight fragments matched within the length range of 100–1500 bases (Figure 4). The very high fraction of matching fragments (80%) showed that the FR primers containing 5′ and 3′ splice signal sequences specifically amplify exons from a given genome.

## Discussion

The above results indicate that multiplexing of exons using FR primers is feasible. The consistent increase in the number of bands observed when the number of fixed primers was decreased and the number of randomized bases was increased in the FR primers suggests that the FGF technique should be generally applicable to multiplex amplify exons from a genome, by using FR primers based on different splice signal sequences. By comparing the same set of amplification products from different samples, sequence differences such as point mutations within the splice signals, or deletion mutations within any of the multiplexed amplified exons, can be revealed by the size shift or the absence of specific fragments. Thus the technology may be broadly applicable for the detection of disease and drug-response gene markers in humans or for the detection of trait associated gene markers in cultivated plants. Because the majority of mutations associated with diseases or traits are within gene functional regions, such as splice signals, promoters, polyA sites, branch sites and exons [7]–[13], this technique has the potential to be highly specific for detecting disease, adverse drug-response and trait-associated mutations. FGF largely avoids the complication of the high frequency of non-specific inter-genomic differences within non-genic regions that is encountered with other techniques, such as the AFLP [17]–[21] and RAPD [22]. FGF thus provides the most complexity reduction focusing specifically on the relevant regions required in the analysis of a large genome such as the human. Recently, array and solution based hybridization techniques have been developed for target sequence enrichment [23]–[25]. The specific PCR based FGF technique may be a viable and cost-effective alternative to array and solution based hybridization enrichment technologies, avoiding the non-specificity problems inherent in hybridization.

With FGF, the number of exonic fragments generated can be controlled by adjusting the number of fixed versus randomized bases within the FR primer. Decreasing the length of the fixed sequence and increasing the number of randomized bases, increases the number of binding sites within the genome, and thus the number of fragments amplified. The FGF method is ideal for analysis of exons from the human genome using automated capillary DNA sequencing equipment (e.g. ABI or APB), as the vast majority of exons (>98%) in the human genome are shorter than 750 bases [16]. FGF is also amenable to high throughput analysis of exons, as automated gel analysis equipment can process as many as 10,000 exons per gel run. Thus, with only 30 runs, all of the 300,000 exons within the human genome can be processed. Alternatively, multiplexed exons (or desired regions) can be pooled and sequenced on an NGS platform using barcoded sequences from multiple patients. The data collection and comparative fingerprint analysis can be automated for comparing the normal and the disease genomes.

With the appropriately designed FR primers, the FGF method can also be applied for the multiplexed genome wide amplification of any targeted regions, such as promoters (e.g, by amplifying the regions between the promoter and the end of the first exon), branch-point sites (e.g., the region between the lariat and the start or the end of the immediately downstream exon), and polyA sites (e.g., the region between the start of the last exon and the polyA site). With one splice signal FR primer directed towards the intron, and a second arbitrary fixed sequence primer, intron sequences adjacent to the exons can be analyzed genome-wide. Genome-wide computational analyses of functional elements such as the promoters should enable the selection of subsets of FR primers for comparison between disease and normal cohorts. FGF can also generate a fingerprint of a genome with any sequence of interest that frequently repeats within the genome. It may be used to clone a library of exons from unsequenced genomes for subsequent sequencing, which may help to identify genes of interest. With arbitrary sequences that may repeat within a genome as the fixed sequences, different variations of FR primers can generate useful fingerprints. This method has the potential for many applications that explore directly the targeted regions of a genome, and may enable fast and relatively inexpensive detection of causal mutations underlying many uncharacterized human illnesses. Additionally, FGF has several applications in microbial genomics such as pathogenic detection and strain identification.

In conclusion, the results demonstrate that genome wide amplification of multiplexed exons and other targeted regions from the human genome is feasible. The high probability that genes bearing causal mutations of diseases and adverse drug effects are contained within these targeted regions gives the FGF technique momentous practical utility in medicine, diagnostics and agriculture. While the current genome wide genotying techniques have not yet uncovered the disease modifying and drug response genes and their relevant causal mutations, the FGF technique may prove to be capable of identifying them effectively. Moreover, this technique is amenable to automated high throughput processing and rapid genome-wide screening of large targeted data. The FGF technique is scalable and can be integrated with the NGS and microarray techniques [26], [27], enabling rapid sequencing of the FGF fragments (e.g., genome wide exons), thereby allowing faster direct comparisons of the targeted regions between the disease and the normal genomes. In addition to its capability to compare fingerprints of functional regions from different genomes for studies including genotypic differences among species, FGF can be an effective front-end target enrichment strategy for NGS platforms, with a variety of biomedical and agricultural applications.

## Methods

### Primer design and preparation

Fixed-Randomized (FR) primers were designed based on the concept described in the text. The fixed base sequence of a given length was synthesized first, and randomized bases were sequentially added at the end of a fixed base sequence with equal concentrations of the A, T, C, and G at each addition. Different FR primers with differing lengths of fixed and randomized sequences were synthesized as required. Primer 3 software [28] was used for primer design.

### Polymerase Chain Reaction

As an example, the DNA fragment representing Exon 7 – Intron 7 – Exon 8 – Intron 8 from the human gene *MYBPC3* was used in this experiment. The forward and the reverse primer sequences and the details of the PCR reactions are as shown in the Table 1. The bases at the 3′ end of the primers were replaced by increasing number of Ns, concomitantly reducing the length of the fixed bases. The PCR experiment was conducted at a Tm of 58°C in a reaction volume of 125 micro-liters. The concentrations of the primers were increased in the different PCR reactions with primers containing Ns as shown in the Table 1. Fifty nanogram of human genomic DNA was used as the template DNA. PCR reaction mixtures contained in a volume of 50 µl: 50 ng of genomic DNA, 100 mM Tris-HCl (pH 8.3), 15 mM MgCl_2,_ 500 mM KCl, 1 mg/ml gelatine, at 20°C, 100–150 pmoles of each primer, all four dNTPs at 200 µM (Roche), respectively, and 1 U of Taq-DNA polymerase (Fisher Scientific). Each PCR reaction was initially denatured at 94°C for 4 min. Cycling conditions (BioRad) were 94°C (30 sec) for denaturation; 55°C (30 sec) for annealing and 72°C (60 sec) for extension. After 38 cycles PCR was terminated by a final synthesis at 72°C for 8 min.

### Computational analysis of the genome-wide distribution of FR primers

The complete procedure involved in computational analysis of the genome is the subject of a separate publication (In preparation). A brief description follows. The human genome sequence was searched for the occurrence of the fixed sequences of a given FR primer pair (e.g., an exon FR primer). The output sequences were matched with the NCBI annotations of exons that contained the computer identified FR splice signals on either or both sides. The fractions of the intron or intergenic sequences containing the computer identified splice signals were also computed. The fraction of the computer amplified exons using a given splice signal FR primer pair that matched with the actual exons within the genome was computed from the above fractions.
